# Concurrent sampling of transitional and coastal waters by Diffusive Gradient in Thin-films (DGT) and spot sampling for trace metals analysis

**DOI:** 10.1016/j.mex.2021.101462

**Published:** 2021-07-21

**Authors:** Philippe Bersuder, Isabelle Amouroux, María Jesús Belzunce-Segarra, Thi Bolam, Miguel Caetano, Inês Carvalho, Margarida Correia dos Santos, Gary R. Fones, Jean-Louis Gonzalez, Stephane Guesdon, Joana Larreta, Barbara Marras, Brendan McHugh, Florence Menet-Nédélec, Iratxe Menchaca, Vanessa Millán Gabet, Natalia Montero, Martin Nolan, Fiona Regan, Craig D. Robinson, Nuno Rosa, Marta Rodrigo Sanz, José Germán Rodríguez, Marco Schintu, Blánaid White, Hao Zhang

**Affiliations:** a Cefas, Centre for Environment, Fisheries and Aquaculture Science, Lowestoft Laboratory, Pakefield Road, Lowestoft, Suffolk NR33 0HT, United Kingdom; b Ifremer, Unit of Biogeochemistry and Ecotoxicology, Rue de l'Ile d'Yeu, 44311 Nantes, France; c AZTI, Marine Research Division, Herrera Kaia Portualde z/g, 20110 Pasaia, Spain; d IPMA, Portuguese Institute of Sea and Atmosphere, Rua Alfredo Magalhães Ramalho, 6, 1495-165 Algés, Portugal; e Centro de Química Estrutural, Instituto Superior Técnico, Av. Rovisco Pais, 1049-001, Lisboa, Portugal; f School of the Environment, Geography and Geosciences, University of Portsmouth, Burnaby Building, Burnaby Road, Portsmouth PO1 3QL, United Kingdom; g Ifremer, LITTORAL, Laboratoire Environnement Ressources des Pertuis Charentais, Avenue de Mus de Loup, 17390 La Tremblade, France; h UNICA, Dipartimento di Scienze Mediche e Sanità Pubblica, Università degli Studi di Cagliari, 09124 Cagliari, Italy; i Marine Institute, Rinville, Oranmore, Galway, Ireland; j Ifremer, LITTORAL, Laboratoire Environnement Ressources de Normandie (LERN), Avenue du Général de Gaulle, 14520 Port-en-Bessin, France; k ITC, Instituto Tecnológico de Canarias, Playa de Pozo Izquierdo, s/n. CP: 35119, Sta. Lucía, Las Palmas, Spain; l DCU Water Institute, Dublin City University, Dublin 9, Ireland; m MSS, Marine Scotland Science, Marine Laboratory, 365 Victoria Road, Aberdeen AB11 9DB, United Kingdom; n Lancaster Environment Centre, Lancaster University, Lancaster LA1 4YW, United Kingdom

**Keywords:** Diffusive Gradient in Thin-films (DGT), Passive sampling, Spot sampling, Metals, Transitional and coastal waters, Monitoring, MONITOOL, EU Water Framework Directive (WFD)

## Abstract

This protocol was developed based on the knowledge acquired in the framework of the Interreg MONITOOL project (EAPA_565/2016) where extensive sampling campaigns were performed in transitional and coastal waters covering eight European countries. It provides detailed procedures and guidelines for the sampling of these waterbodies by concurrent collection of discrete water samples and the deployment of Diffusive Gradient in Thin-films (DGT) passive samplers for the measurement of trace metal concentrations. In order to facilitate the application of this protocol by end-users, it presents steps to follow in the laboratory prior to sampling campaigns, explains the procedures for field campaigns (including *in situ* measurement of supporting parameters) and subsequent sample processing in the laboratory in preparation for trace metal analyze by inductively coupled plasma-mass spectrometry (ICP-MS) and voltammetry. The protocol provides a systematic, coherent field sampling and sample preparation strategy that was developed in order to ensure comparability and reproducibility of the data obtained from each project Partner in different regions.

• Standardization of the concurrent sampling of transitional and coastal waters by DGT passive samplers and spot sampling.

• Robust procedures and tips based on existing international standards and comprehensive practical experience.

• Links to demonstration videos produced within the MONITOOL project.

## Graphical abstract

**Figure:**
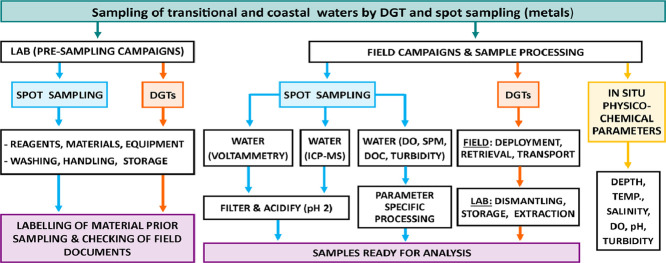

Specification table

**Table:** 

Subject Area	Environmental Science
More specific subject area	Water Quality Monitoring by Passive Sampling
Method name	Concurrent sampling of transitional and coastal waters by Diffusive Gradient in Thin-films (DGT) and spot sampling for trace metals analysis
Name and reference of original method	This protocol is based on guidance described in: [4] ISO 5667-9:1992 Water quality — Sampling — Part 9: Guidance on sampling from marine waters [5] ISO 5667-23:2011. Water quality – Sampling - Part 23: Guidance on passive sampling in surface waters. [9]. General Guide for using DGT in Waters [WWW Document]. DGT Res. Ltd , Skelmorlie, Lancashire, UK. URL https://www.dgtresearch.com/wp-content/uploads/2020/09/waters.pdf (accessed 2020.9.25).
Resource availability	N/A

## Method details

### Background

MONITOOL (https://www.monitoolproject.eu) is a European Interreg Atlantic Area project whose objective is to provide a robust database of dissolved and labile metal concentrations in transitional and coastal waters. The evidence will be used for the purpose of adapting existing Environmental Quality Standards (EQS; 0.45 µm-filtered) for diffusive Gradients in Thin-films (DGT) passive sampling devices (EQS_DGT_). This will allow a more accurate evaluation of the chemical status of waters under the EU Water Framework Directive (WFD) [3]. The overarching objective of MONITOOL is to underpin acceptance of DGTs for the assessment of the chemical status of marine waters in compliance with EU WFD requirements.

To this end, a bi-seasonal survey program consisting of the concurrent deployment of DGT passive sampling devices and collection of discrete spot water samples was performed by nine research organizations (Arrantzuarekiko Zientzia eta Teknika Iraskundea (AZTI), Centre for Environment, Fisheries and Aquaculture Science (CEFAS), Dublin City University (DCU), Institut Français de Recherche pour l'Exploitation de la Mer (IFREMER), Portuguese Institute of Sea and Atmosphere (IPMA), Instituto Tecnológico de Canarias (ITC), Marine Scotland Science (MSS), Scottish Environment Protection Agency (SEPA) and Università degli Studi di Cagliari (UNICA)). The monitoring campaigns covered eight countries across the Atlantic region, from the Canary Islands (Spain) to the Scottish Highlands & Islands, as well as Sardinia (Italy) in the Mediterranean region. A common laboratory and sampling protocol was developed to standardise a series of guidelines/methodologies that were followed by all participating partners in order to ensure the comparability and reproducibility of data obtained from each Partner in different regions. The protocol also provides indications of sample processing for the subsequent analysis of trace metals by inductively coupled plasma mass spectrometry (ICP-MS) (typically used for compliance monitoring analysis) or voltammetry. Guidance for the collection of discrete water samples for the measurement of supporting parameters, such as suspended particulate matter (SPM), dissolved organic carbon (DOC), dissolved oxygen (DO), pH, temperature and salinity, is also provided. General guidance on sampling from marine waters such as EN ISO 5667-9 [4] and ISO 5667-23 [5] should be consulted in conjunction with this protocol.

Five MONITOOL videos were produced by IFREMER (France) to support this sampling protocol: ‘MONITOOL field Sampling - A demonstration with Jean Louis Gonzalez’ and four tutorials on the use of DGTs (https://www.monitoolproject.eu/multimedia/videos).

## Reagents and materials

### Reagents

(i) *DGT*

Water, ultrapure, type I or better (≥ 18 MΩ•cm resistivity).

(ii) *Spot Sampling*

Nitric acid (HNO_3_), 69%, ultrapure grade.

(iii) *Generic*

Nitric acid (HNO_3_), 69%, analytical grade.

Water, deionised.

### Materials

(i) *DGT*

The passive sampler selected for the MONITOOL project was the LSNM-NP Loaded DGT device for cationic trace metals in waters consisting of a standard DGT plastic holder with a polyethersulphone filter membrane (0.45 µm pore size), 0.8 mm agarose cross-linked polyacrylamide (APA) diffusive gel and Chelex® binding layer (DGT® Research Ltd, Lancaster, UK), all from the same manufacturing batch. This DGT device can be used to measure up to thirty metals, including Cd, Co, Cu, Fe, Mn, Ni, Pb and Zn.

Gloves, powder-free (uncoloured if possible).

Clamps (*), uncoloured (e. g., cable tie).

Bags (*), plastic.

Netting (*), uncoloured or white (optional).

Tweezers/forceps (*), plastic (white if possible).

Micro-centrifuge tubes (*), plastic, 1.5 mL and 5 mL.

Pipette tips (*), 100 µl, 1 mL and 5 mL.

(ii) *Spot water Sampling*

Bottles (*), 500 mL, high density polyethylene (HDPE), high performance rated, translucent, narrow or wide neck, with screw cap (*).

Bottles (*), 250 mL, borosilicate glass, clear or amber, with polytetrafluoroethylene (PTFE) sealed lid (*) (e.g., DURAN® PURE bottles and Premium screw caps).

For the preparation of samples for trace metals analysis by voltammetry:

*Digi*FILTER™ (*), 0.45 µm, Teflon® membrane, 50 mL tubes (SCP SCIENCE, Quebec, Canada).

*Digi*TUBEs™ (*), 50 mL or 100 mL, Non RackLock w/caps (SCP SCIENCE, Quebec, Canada).

Syringes, plastic, 50 mL.

For the preparation of samples for trace metal analysis by ICP-MS and for SPM determination:

Filters (*), polycarbonate, pore size 0.4 μm, 47 mm diameter (e.g., Whatman® Cyclopore® membrane).

NOTE: Material with (*) must be cleaned as described below:

Analytical grade nitric acid (69%) must be used to make up a 10% (v/v) aqueous HNO_3_ bath, where the material to be used in the laboratory and sampling campaigns will be immersed for at least 4 h, and up to overnight. Materials must be rinsed with ultrapure water, dried under a laminar flow hood (see *Equipment* below) and stored in cleaned and sealed plastic bags until utilisation. It is not recommended to carry out the cleaning of the *Digi*TUBEs™ and *Digi*FILTER™ three months or more before their use, due to the residual acid remaining in the tubes/filters.

*Equipment:*

(i) *DGT*

Ropes, weights, etc…

Cool box.

Ice packs.

Plastic screwdriver (or metal screwdriver covered with plastic or glove).

Laminar flow hood, positive pressure.

Pipette, variable volume: 100 µl, 1 mL and 5 mL.

(ii) *Spot water sampling*

Water sampler: direct sampling using grab samplers with HDPE bottles is recommended. Discrete, subsurface seawater samples can also be collected using devices such as Niskin bottles, handheld water samplers or remote automatic samplers.

(iii) *Generic*

Independent or multiparametric probes for *in situ* measurement of temperature, pH, salinity, dissolved oxygen and turbidity. The pH meter must have a minimum resolution of 0.1 and minimum accuracy of ±0.2. The dissolved oxygen probe must be suitably calibrated, e.g., to the Winkler method [1]. Some of these parameters (e.g., turbidity, salinity, dissolved oxygen) can also be measured in the laboratory.

All sensor equipment used in the sampling campaigns is calibrated at least to the frequency provided in Table 1.

**Table 1:** Calibration frequency for sensor equipment.

Parameter to be measured	Frequency
Dissolved Oxygen_,_ 100% Saturation	Each Day of Use
Depth Zero	Each Day of Use
pH	Each Day of Use
Salinity	Monthly
Temperature	Annual
Turbidity	Each Day of Use

## Procedure

This section provides the procedures for discrete spot water sampling (A), physico-chemical data recording (B) and DGT sampling (C) and subsequent sample processing (up to instrumental analysis) as developed and applied by MONITOOL project Partners. Water spot sampling is performed prior to the measurement of physico-chemical parameters (temperature, salinity, dissolved oxygen, pH) and to the deployment of DGT passive samplers.

A. SPOT WATER SAMPLING

This involves collecting discrete subsurface water samples in transitional and coastal waters for the measurement of the concentrations of the EU Water Framework Directive priority metals Pb, Ni, Cd, as well as Al, Co, Cu, Fe, Mn and Zn. The procedure detailed is essential to avoid contamination during sampling of the water samples to be subsequently analysed for trace metals determination by either ICP-MS or voltammetry.

Direct sampling into pre-cleaned bottles should be done as best practice. In the case of sampling using other water sampler devices such as a Niskin bottle, sampling should be done using clean silicon tubing; under no circumstances should Tygon® tubing be used as it is a source of contamination [2]. Avoid touching the Niskin bottle sampling port unless using trace metals clean sampling equipment (tubing) and gloves. Grease should never be allowed to come in contact with the sample nipple. Gloves must be worn during sampling.

### Sampling frequency

At tidal estuarine sites, spot sampling should be performed twice a day (i. e. at high and low tide) for each day of the DGT deployment period. Ideally sampling should be planned in a way to minimise salinity variation.

In other transitional waters (with reduced tidal influence) and coastal sites, daily sampling is ideal although weather conditions might prevent this from happening. Therefore, sampling should be done once a day, on day 0 (i.e., at DGT deployment time), day 2 (i.e., 48 h after deployment) and day 4 (i.e., approximately 4 × 24 h after deployment, that corresponds to the retrieval time of DGTs).

### Water sampling procedure

1. Deploy water sampler at the same depth as planned for the installation of DGTs and fill up the water sampler. Discard the water from the sampler and repeat this activity two more times.
2. Once the water sampler has been rinsed twice, collect the water sample sequentially for:
  - Dissolved oxygen (if DO is not measured by a sensor *in-situ*). Note that samples for DO must be collected and treated first due to rapid loss of integrity with time. If the samples are to be analysed in a laboratory, special bottles (i.e., Winkler bottles or calibrated glass stoppered bottles) and chemical preservation are required to store the samples.
  - Record the *in-situ* temperature and salinity at the time of sampling for DO calculation.

If trace metals are to be analysed by voltammetry: follow the procedure (3-11) below for sample preservation. Filtration must be carried out as soon as possible, preferably on-site, and high-quality powder free gloves must be worn for the whole filtration process: If *on-site* filtration is not practical due to the weather conditions, sampling space limitations, etc., then store the sample in a cool box with ice packs and filter the sample as soon as possible (i.e., preferably within 4 hours of sampling). Precautions should be taken to minimize ambient contamination, and these steps should be done under a protective bag or a laminar flow hood if in the laboratory.

3. Rinse the 500 mL HDPE bottles (with closed bottle cap) three times with the seawater collected in the water sampler and fill it up.
4. Rinse DigiTUBE™ no. 1 twice with an aliquot of seawater from the HDPE bottle and discard the water. Fill in *Digi*TUBE™ no. 1 again with 50 mL (or 100 mL depending on *Digi*TUBE™ volume size) of seawater from the HDPE bottle.
5. Assemble DigiTUBE™ no. 1 with the *Digi*FILTER™ and screw in *Digi*TUBE™ no. 2 at the other side. Ensure that the entire assembly has a firm seal.
6. Invert the DigiTUBE™-DigiFILTER™ assembly so that the water sample is on top. Connect the syringe into the hole of the DigiFILTER™, remove the red insert from the *Digi*Filter™ and start the filtration. Discard the first 10 mL of filtrate. Filter 50 mL/100 mL of seawater sample into *Digi*TUBE™ no. 2, remove it and close it tight with its respective lid. Repeat steps 5 and 6 with *Digi*TUBE™ no. 3. If filtration is too slow, then the *Digi*FILTER™ might need to be replaced.
7. Acidify the filtrates to pH 2 by adding 0.035 mL of HNO3 (69%, ultrapure grade) to each 50 mL of seawater sample (or 0.070 mL of HNO3 to 100 mL of seawater) just after filtration.
8. After capping the DigiTUBEs™, seal the tubes if necessary.
9. Keep the acidified samples upright and refrigerated (4 °C). Under these conditions, the samples are stable for several weeks.
10. Sample blanks for voltammetry: for each site, on days 0 and 4, prepare voltammetry blanks by filtering 2 × 50 mL ultrapure water (or 100 mL depending on *Digi*TUBE™ volume size) through a pre-cleaned *Digi*FILTER™ (steps 5-6) and following steps (7-9) for the acidification and conservation of collected samples.
11. Sample filtrates can be stored until the end of the DGT sampling campaign and sent in a cool box with ice packs to the analytical laboratory for the determination of trace metals by voltammetry as soon as practicable.

If trace metals are to be analysed by ICP-MS: follow the procedure (12-13) below for sample preservation:

12. Collect ca 100 mL of seawater sample in a pre-cleaned and labelled HDPE bottle. This must be collected at the same time as for the voltammetry sample; therefore, the remaining water collected in the 500 mL HDPE bottle (step 3 above) can be used for this. Store the sample in a cool box with ice packs before sending to the laboratory.
13. Sample blank for ICP-MS: for each site, at days 0 and 4, bring one empty 500 mL HDPE bottle to the field and expose the blank bottle to the ambient air by opening it during the time of collection of water samples. The bottle for the sample blank can be kept empty to facilitate transport to the analytical laboratory. Close the bottles after the spot sampling is completed and transfer the empty blank bottles along with the seawater samples to the analytical laboratory in a cool box. Once in the analytical laboratory, the bottles are opened during the sample filtration procedure and closed after finishing.

Sampling for dissolved organic carbon (DOC) analysis: follow procedure (14-17)

14. A 250 mL pre-cleaned DURAN® PURE glass bottle should be rinsed 3 times with the water collected from the water sampler.
15. Fill the rinsed bottle up to the brim (with no head space) with the water sample.
16. Cap the bottle tightly, clearly label it and store cool in the dark until analysis.
17. Blank sample for DOC consists of deionised water from the analytical laboratory.

Sampling for Suspended Particulate Matter (SPM) determination: follow procedure (18-21)

18. A 500 mL pre-cleaned HDPE bottle should be rinsed 3 times with the water collected from the water sampler.
19. Collect 500 mL of water sample into the rinsed bottle.
20. Cap the bottle tightly, label it and store it in a cool box for transport to the analytical laboratory.
21. Blank sample for SPM consists of deionised water from the analytical laboratory.

Sampling for turbidity analysis: follow procedure (22-23)

22. If turbidity is not directly measured *in-situ* by sensor, collect 100 mL of seawater for turbidity analysis and refrigerate until analysis, which must be carried out within 48 h of sampling due to its instability.
23. The blank sample for turbidity consists of deionised water from the analytical laboratory.

B. PHYSICO-CHEMICAL DATA RECORDING

*In-situ* physico-chemical parameters of water must be recorded with a calibrated instrument at the DGT sampling depth at each site visited and at each sampling time.

The physico-chemical parameters to be measured are: depth, temperature (°C), salinity, dissolved oxygen (% and mg/L) and pH.

Discrete water samples are to be taken for the determination of other supporting physico-chemical parameters, including SPM, DOC and turbidity by nephelometry determination (although turbidity can also be measured *in-situ* or under laboratory conditions by sensors). Procedures for sampling are provided in section (A) above.

C. DGT BASED PASSIVE SAMPLING

The following guidelines for general DGT handling, storage and deployment are based on the “General Guide for using DGT in Waters” published by DGT® Research Ltd [9]. The analysis of accumulated trace metals was performed by ICP-MS.

Please note that under perfect sink and steady-state conditions, the time-dependent accumulation of metals in DGTs is expected to be linear until saturation of the binding layer [10] (capacity limitations are not usual in conventional 3–21 days deployments). However, non-linear accumulation of metals can occur due to competition between dissolved species for the binding sites, especially when approaching the effective capacity of the binding resin [6, 7]. Such conditions might for example occur in highly contaminated sites. Users should therefore consider a shorter deployment time or a thicker diffusive gel disc when deviation from linearity is expected. Effects from biofouling are considered to be minimal for periods of less than seven days deployment [8].

### General DGT handling guidelines

DGT devices are supplied in sealed, clean plastic bags containing ca 0.5 mL of 0.01M NaNO_3_ (or 0.01M NaCl) solution. In order to prevent contamination of the DGT units, clean handling procedures should be adopted during their deployment and recovery, and subsequently during the sample processing steps. Direct contact with the DGT devices must be minimised:

• Do not open or remove DGT units from the sealed plastic bag until immediately (minutes) prior to assembly or deployment.
• Always wear powder-free, preferably uncoloured gloves when handling DGT units to avoid contamination.
• Do not touch or let anything come into contact with the white filter membrane present in the exposition window of the DGT device.

### DGT pre-deployment storage

• Store the DGT units under refrigerated conditions (4 °C), avoid freezing as performance could be affected.
• Check the DGT units about once a week to ensure that moist conditions are maintained. Add ca 0.5mL of trace metal clean 0.01M NaNO_3_ (or 0.01M NaCl) solution if necessary.

### DGT assembly

**Fig. 1:**
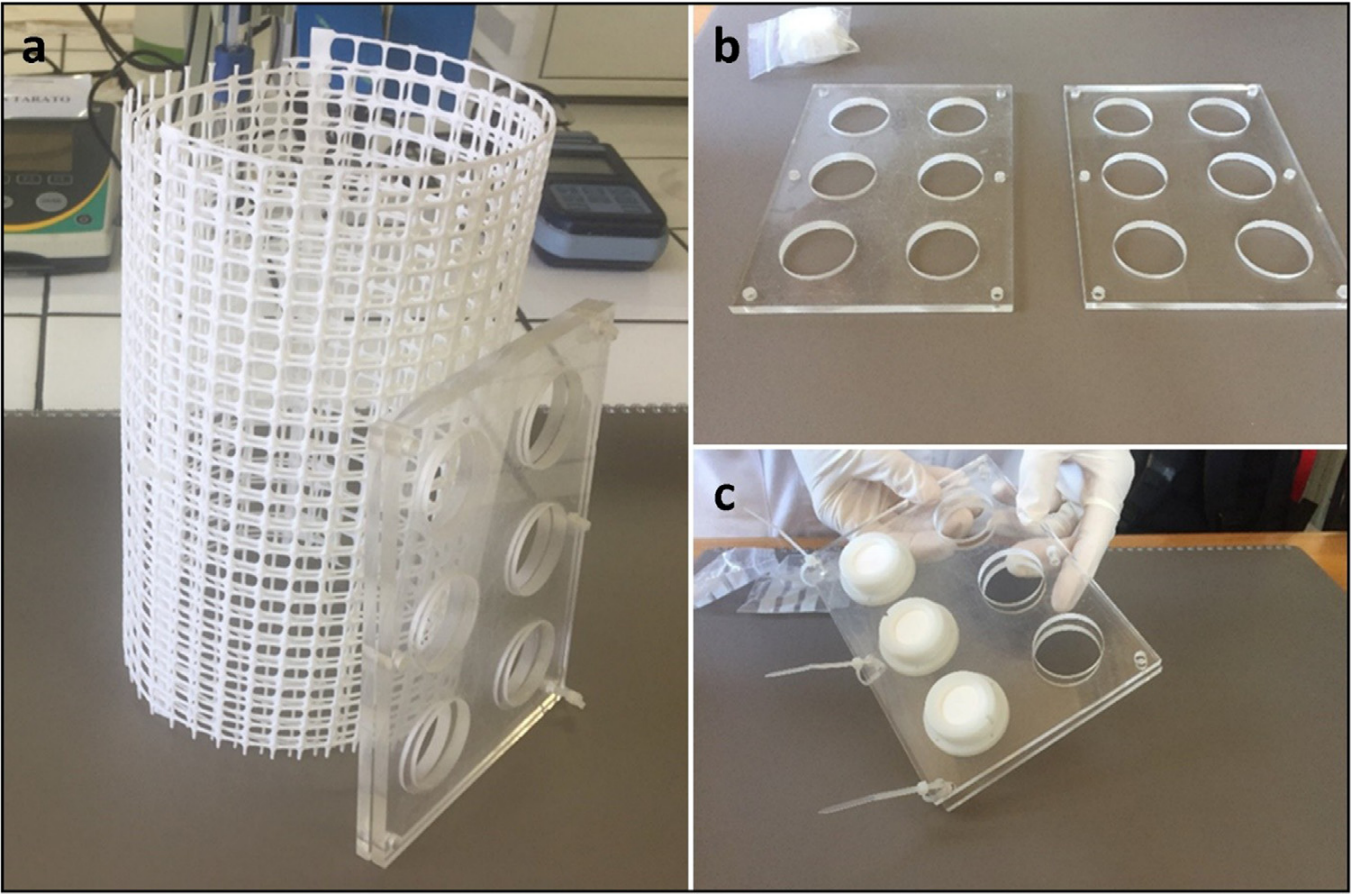
Structure for the deployment of DGTs: (A) DGT holder and net; (B) detailed photograph of the DGT holder; (C) placing of DGTs in the DGT holder (Source: UNICA).

Prior to deployment, DGT devices are assembled in a clean laboratory environment, preferably under a laminar flow cabinet or in a plastic bag to avoid contamination from air exposure. DGT devices are loaded onto a DGT-holder and protected with a net (Fig. 1), if necessary, to prevent damage from side impact and possible interference by fish and other organisms present in the deployment zone. Ideally DGTs should be mounted onto the holder in the laboratory and the whole assemblage (with or without the net) must be stored as described below, in sealed plastic bags and kept refrigerated before deployment. Pre-assembly of the nets can be performed in the laboratory to speed up the DGT deployment and to limit exposure of devices to ambient air. Laboratory Blank DGTs and Field Blank DGTs are kept inside their individual plastic bags (not mounted onto holders).

Open the individual bags and expose DGT Field Blanks on a clean surface for the entire duration of the holder/DGT assembly (i.e., simultaneously exposed while manipulating the field DGTs before deployment). After exposure, the DGT Field Blank is transferred back in its original plastic bag and stored refrigerated as described above. DGT Field Blanks are further exposed in the field during the DGTs deployment and retrieval processes and in the laboratory during the holder/DGT disassembly.

### Transport to deployment site

**Fig. 2:**
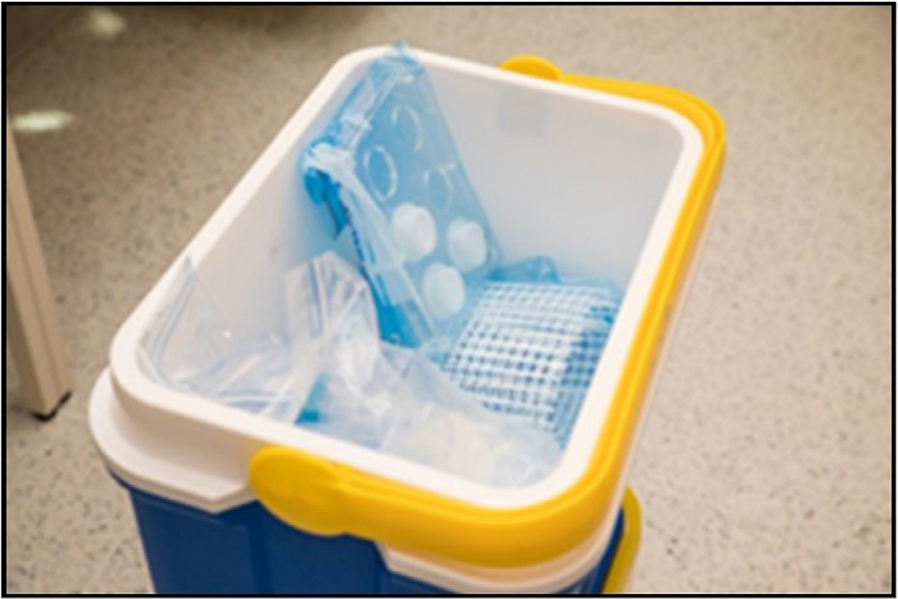
Double bag DGT assembly and other needed material in cooler box (Source: ITC).

Sealed plastic bags, containing each DGT assemblage (DGT, holder and net), and DGT Field Blanks must be transported to the field in a clean cool box/polystyrene box with ice packs (Fig. 2).

### Designated deployment structure

**Fig. 3:**
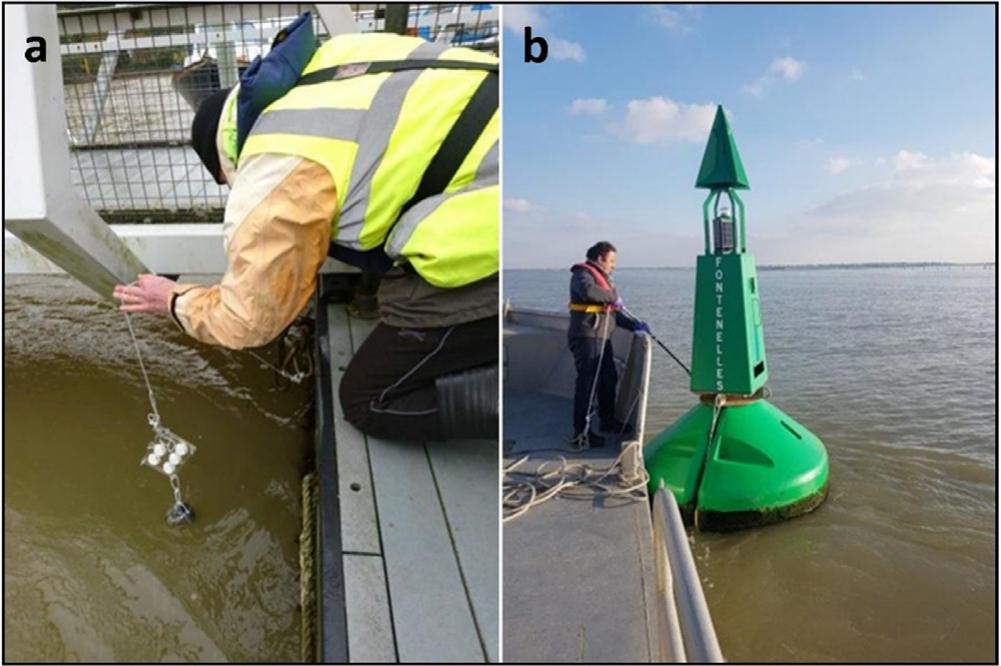
DGT deployment structures used in MONITOOL a) Fal Estuary, UK (Source: CEFAS) and b) Fontelles, France (Source: IFREMER).

**Fig. 4:**
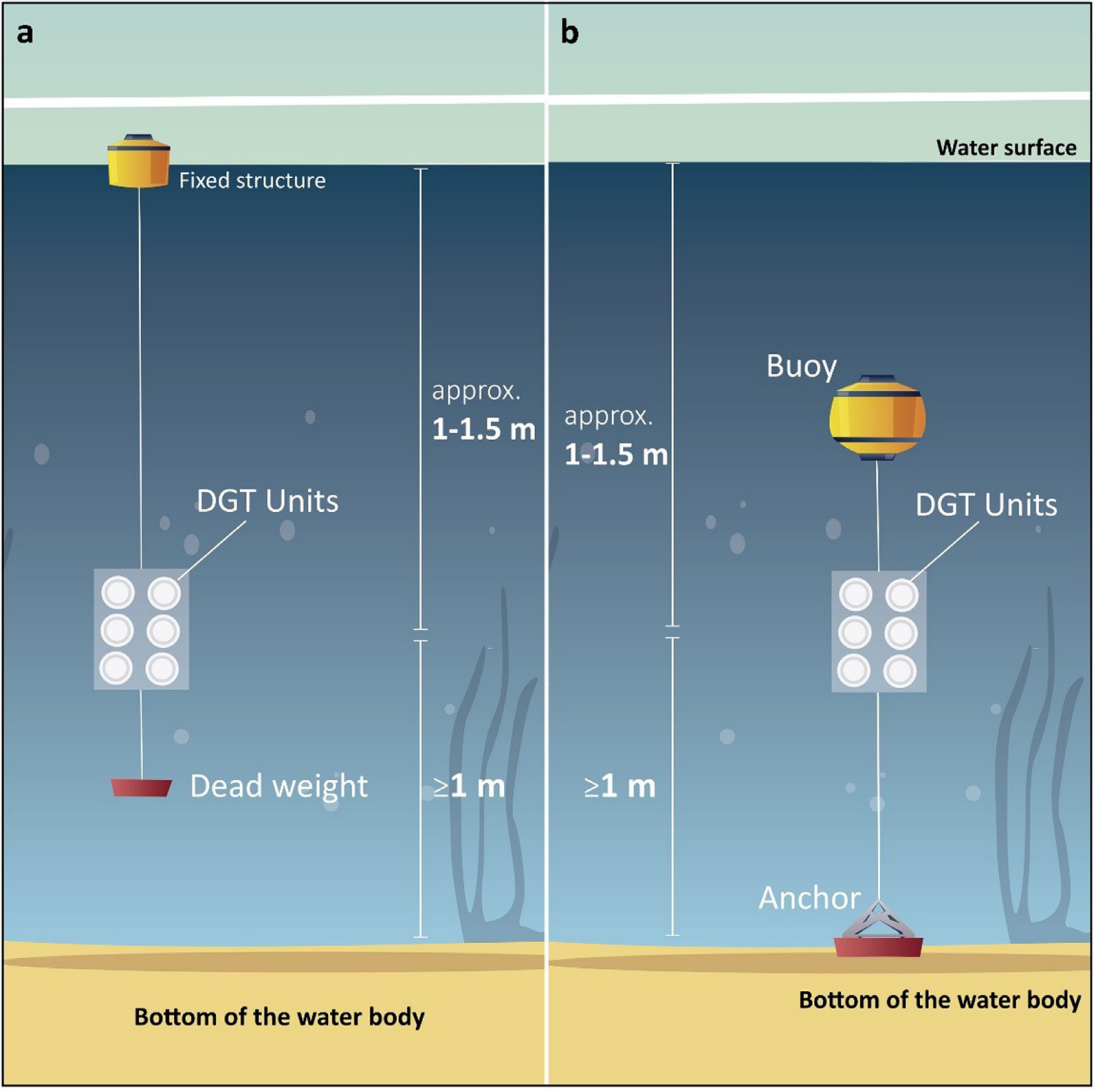
Designated structure options for DGT deployment a) left and b) right (Source: ITC).

The easiest deployment system consists of the use of moorings, buoys or other fixed structures (Fig. 3) where the DGT assembly is attached with a rope presenting a weight at the end (Fig. 4a). Another option is to place the DGT assembly between a weight resting on the bottom and a buoy in order to keep the DGTs at the desired height (Fig. 4b).

### Sampling duration

Optimum deployment time depends on the dissolved metal concentrations in water and the quantification limits of the analytical technique. Deployment times between 3 and 21 days might be appropriate, if site-specific characteristics do not favor any significant biofouling development on DGT sampling surfaces. If metal concentrations are expected to be very low, as in an offshore marine environment, and there is no indication of biofilm growth on the surface of the devices, longer deployment times may be appropriate.

The protocol used during MONITOOL sampling campaigns fixed the DGT deployment time to four days (i.e., 4 × 24 h).

### DGT deployment

24. Wearing powder free gloves, remove the DGT assemblages from their plastic bags and attach the DGT holder onto the designated structure. For example, the DGT holder can be attached to a rope which has got a weight at the end (Fig. 4). The rope is then secured onto the mooring buoy or fixed structure (away, as much as possible, from any metallic structure).
25. Ensure that the DGTs are deployed in flowing (or moving) water, but avoid excessive turbulence, particularly bubbles.
26. Deploy the DGT devices immediately, ideally at a depth of 1–1.5 m from the surface and at least 1 m above the seabed. In shallow areas, ensure that the DGT devices are fully immersed if depth is <1 m and at least 0.3–0.5 m above the seabed. However, sampling site characteristics must be considered for the selection of the most suitable sampling depth (e.g., in harbours, choosing a greater depth might guarantee a reduction in potential high variability associated to shipping).
27. Ensure that the sampling windows of DGTs will remain fully immersed during the deployment period.
28. Simultaneously, expose the DGT Field Blank on a clean surface (Fig. 5) until the DGTs assemblage is submerged in the water. Immediately after deployment of the DGT holder, put the DGT Field Blank back in its original plastic bag and store refrigerated until the DGT retrieval day.

**Fig. 5:**
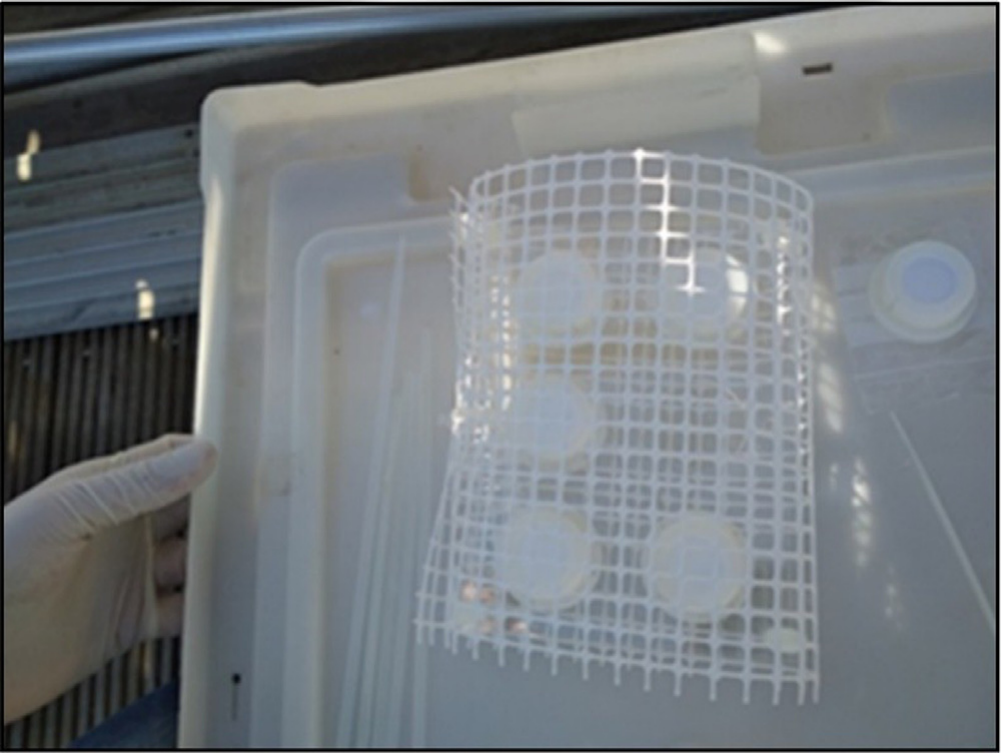
DGT exposure of the DGT Field Blank at Tagus location in Portugal (Source: IPMA).
29. **Rec**ord accuratel**y the time of deployment to the nearest minute**.
30. Record the depth of deployment. Keep the original plastic bags (clearly labelled and sealed) to store the DGT assemblages at the retrieval stage.
31. Record water temperature during the deployment time. If the temperature variation during the deployment period is within ± 2 °C, a mean value (or start and end temperature) will suffice. If the variation is greater, ideally the mean temperature should be obtained from an integrated record of temperatures (e.g., using data loggers).

### DGT *retrieval and transport*

32. Field Blank exposure: simultaneously to DGT retrieval, expose the DGT Field Blank (previously exposed during the deployment) on a clean surface for the entire duration of the retrieval process. DGT Field Blanks are returned to their original plastic bags and kept in a cool box with ice packs for transport to the laboratory.
33. Remove the DGT holder unit from the deployment structure and take it out of the water wearing gloves (powder free, uncolored if possible), taking care not to touch the DGTs’ filter membrane. **Record the retrieval time to the nearest minute**.
34. Rinse the DGT holder immediately after recovery with water from the site by direct immersion of the device (e.g., from the boat, from the dock, …) and by shaking the device underwater several times (can be done manually or without detaching the holder from the mooring). Alternatively, rinse the DGT holder and DGT units with a stream of uncontaminated distilled/deionised water from a clean wash bottle.
35. Shake off obvious surface water (do not dry).
36. Place the DGT holder and DGT devices in their original plastic bag and seal with minimum air space. Label the bag with the sampling location and store it in a cool box with ice packs to transport back to the laboratory.
37. Record the temperature of the water at the retrieval time.

### DGT dismantling and preservation

The following steps should be carried out in the laboratory, under a laminar flow hood or in a plastic bag, wearing powder-free gloves:

38. Expose the DGT Field Blank (previously exposed during the deployment and retrieval processes) on a clean surface for the entire duration of the holder dismantling, then return the DGT Field Blank back in its original plastic bag.
39. Remove the individual DGT devices from its holder unit and put them in individually labelled plastic bags (using the plastic bags provided by DGT® Research Ltd when the DGT devices were initially stored in individual plastic bags). Alternatively, if possible, go to step 42 for sample treatment without further storage.
40. Double bag the three exposed DGT devices and the DGT Field Blank. Store in a refrigerator (4 °C) until the start of the elution process.
41. DGT Laboratory Blanks consist of DGTs that are **not exposed** at any time. A minimum of three DGT Laboratory Blanks are recommended to allow the detection of anomalous values (such as from metals contamination resulting from the manufacturing process).

### DGT dismantling and pre-analysis extraction

Powder-free gloves must be worn at all time. The procedure must be conducted in a clean, positive pressure laminar flow hood. In MONITOOL, the procedure of dismantling DGT devices deployed by all Partners was centralized in a single laboratory (IFREMER Toulon, France). Follow the following order for the dismantling of various DGT devices: (i) DGT Laboratory Blank, (ii) DGT Field Blank and (iii) deployed DGT devices (from least to highest expected contamination).

42. To retrieve the resin gel, insert a plastic screwdriver (or metal screwdriver covered with plastic or glove) into the grove in the cap and twist. The cap will break at the weak point (Fig. 6 a). (Note: In the case of step 41 failure, use a clean tweezers to break the white filter membrane, pull out the gels directly and retrieve the resin gel from the bottom. Make sure the tweezers are clean before touching the resin gel).

**Fig. 6:**
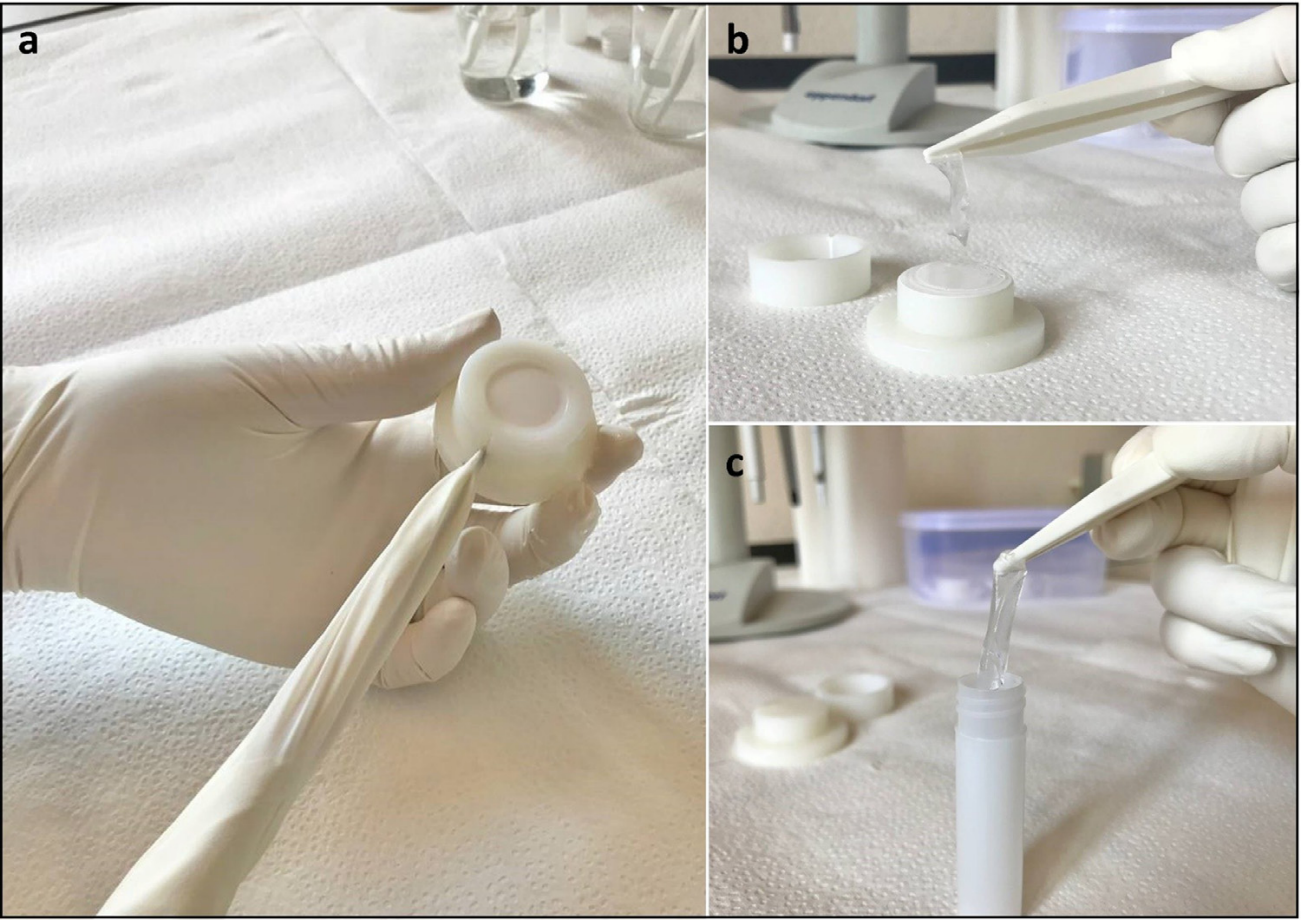
a) DGT opening; b) Chelex® peel off; c) Chelex® being placed in a sample tube (Source: UNICA).
43. Remove the broken cap and then peel off the filter and diffusive gel layer to reveal the bottom resin-gel layer. Alternatively, turn over the complete assembly and take only the thin layer of Chelex® that is then left on top (Fig. 6 b).
44. Place the resin gel, with plastic tweezers, in a clean sample tube and add 1 mL of 1M HNO_3_ solution (Fig. 6 c).
45. Ensure that the resin gel is fully immersed in the HNO_3_ solution. Leave at least 24 h at room temperature. (Note: for urgent analysis, elute gel for at least 2 h on a shaker).
46. Once the elution time has expired, remove the Chelex® resin from the tube. Pipette an aliquot from the sample tube into a new clean tube and dilute at least 10 times with ultrapure water to avoid possible minor saline matrix issues during the analysis. (Note: To avoid any broken gel pieces or resin getting into the diluted solution, pipette from the top of the sample tube).
47. Store solutions refrigerated (4 °C) until trace metals analysis by ICP-MS (or similar technique).

## Conclusions

This protocol provides standard methods and guidelines for the concurrent sampling of transitional and coastal waters by DGT passive samplers and spot sampling for trace metals analysis. Additionally, detailed indications of the subsequent processing of water samples and DGT devices in preparation for the instrumental analysis are given. This protocol was applied by nine international Partners involved in the MONITOOL project and provides guidelines to minimise the risk of contamination during sampling and sample processing and to ensure the comparability and reproducibility of the data obtained at different sampling sites.

## Declaration of Competing Interest

The authors declare that they have no known competing financial interests or personal relationships that could have appeared to influence the work reported in this paper.
